# An original design of remote robot-assisted intubation system

**DOI:** 10.1038/s41598-018-31607-y

**Published:** 2018-09-07

**Authors:** Xinyu Wang, Yuanfa Tao, Xiandong Tao, Jianglong Chen, Yifeng Jin, Zhengxiang Shan, Jiyang Tan, Qixin Cao, Tiewen Pan

**Affiliations:** 1 Department of Thoracic Surgery, Eastern Hepatobiliary Surgery Hospital affiliated to Second Military Medical University, Shanghai, China; 2 Hefei University of Technology, Hefei, Anhui China; 3 The Graduate School of Fujian Medical University, Fuzhou, Fujian China; 4 Department of Orthopedics, Northern Jiangsu People’s Hospital Affiliated to Yangzhou University, Yangzhou, Jiangsu China; 5 Shanghai Jiaotong University, Shanghai, China

## Abstract

The success rate of pre-hospital endotracheal intubation (ETI) by paramedics is lower than physicians. We aimed to establish a remote robot-assisted intubation system (RRAIS) and expected it to improve success rate of pre-hospital ETI. To test the robot’s feasibility, 20 pigs were intubated by direct laryngoscope or the robot system. Intubation time, success rate, airway complications were recorded during the experiment. The animal experiment showed that participants achieved a higher success rate in absolute numbers by the robot system. In summary, we have successfully developed a remote robot-assisted intubation system. It is promising for RRAIS to improve the success rate of pre-hospital ETI and change the current rescue model.

## Introduction

Since the Operation Lindbergh was performed in 2001, telecommunication technique, which demonstrated the feasibility of remote operation in this event, has played a significant role in medicine^1^. A model of combining network with medicine has unique features, which goes beyond the obstacle of long distance and enables people to have access to the latest medical resources. As a result, telemedicine, such as remote consultation and telesurgery, have developed fast in the last decade. We can envisage that telemedicine has a wider application value at places inconvenient for people to reach, such as earthquake zones, aircraft carriers, and nuclear polluted areas.

Endotracheal intubation (ETI) is one of the most commonly performed procedures in first aid and surgery. However, some emergency medical technicians (EMTs) who lack qualifications are unable to perform pre-hospital ETI by themselves. Inexperienced EMTs contribute to more unsuccessful pre-hospital intubations^2^. In addition, success rate of pre-hospital intubation differs widely for different manipulators’ experience. Intubation success rate of non-physician clinicians is lower than physicians^3^.

In order to solve the problem, we originally designed and established a remote robot-assisted intubation system (RRAIS) to fulfil remote ETI. RRAIS can divert intubation job from front-line EMTs to rear anesthesiologists and other specialists who have qualifications and richer experience. It is possible for medical specialists away from the accident sites to perform ETI in the future.

In order to test the robot’s functions, we apply it on pigs and observe the outcomes of intubation.

## Methods

### Ethics statement

The Ethics Committee of Laboratory Animal Center of Second Military Medical University (No. 10 Zhengtong Road, Yangpu District, Shanghai, China), approved this study (Date received: 25/12/16, Date approved: 30/12/16). We confirm that all methods were carried out in accordance with relevant guidelines and regulations. We confirm that all experimental protocols were approved by a named licensing committee. All procedures performed in studies involving animals were in accordance with the ethical standards of the institution or practice at which the studies were conducted.

### Participants

From April 2017 to January 2018, 10 fifth-year undergraduate medical students from Second Military Medical University, who had insufficient experience with ETI (They had performed less than 10 intubations on patients before), were invited to participate in the study. An anesthetist with eight years of working experience from Eastern Hepatobiliary Surgery Hospital and several laboratory workers were enrolled in the study. Informed consent was obtained from all individual participants included in the study before enrollment.

### Animals and anesthesia

Twenty Bama miniature pigs with average weight of 23.2 Kg and 8–10 months of age, were bought from Shanghai Jiagan Biotechnique Co, Ltd. All pigs were preoxygenated for 5 minutes via a tight-fitting facemask and were placed in dorsal recumbency. Atropine 0.05 mg/kg was injected to decrease the secretion of spittle 30 minutes before induction. A drop of 2 ml 2% lidocaine was applied on posterior pharyngeal wall.

Anesthesia was induced by intramuscular injection of 0.5 mg/kg midazolam and intraperitoneal injection of 2 ml/kg 3% pentobarbital sodium. 2 mg/kg of propofol and 1 μg/kg of remifentanil were administrated through ear veins. No neuromuscular blocking agents were used. Anesthesia was maintained by a continuous infusion of 3 mg/kg/h propofol and 0.5 μg/kg/min remifentanil.

### Mechanical design

**Figure 1:**
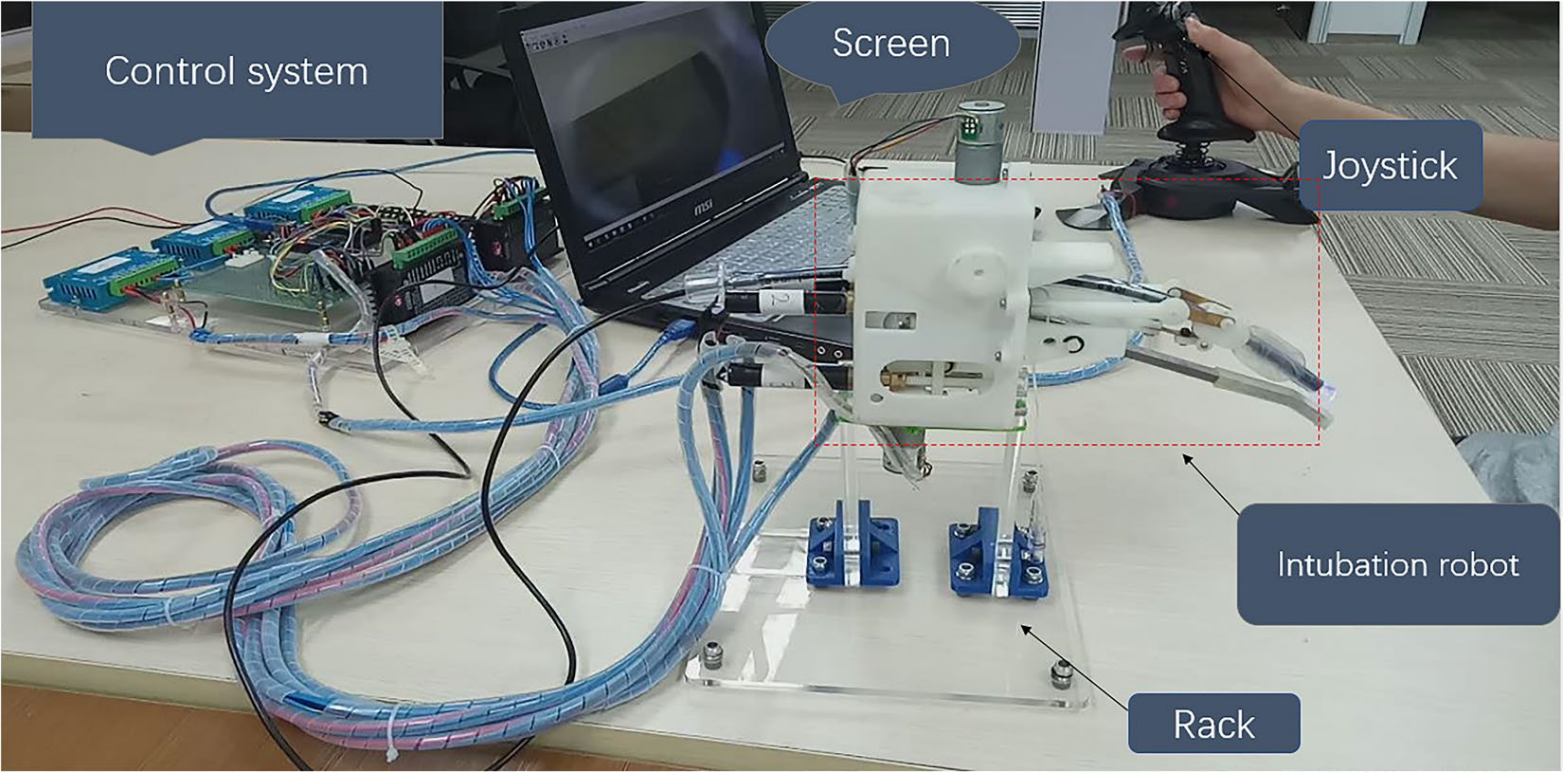
Four components of RRAIS. Including one intubation robot, one control system, one laptop, and one joystick.

A self-made robot system by us named RRAIS were used in the study. As is shown in Fig. 1, RRAIS is composed of four main components: one intubation robot (self-made), one control system (self-made), one laptop (MSI GE62 6QC), and one joystick (Cyborg V.1, MAD CATZ). The intubation robot, mainly including tongue depressor, posture mechanism, and feeding mechanism, was the most important part of the robot system.

### Tongue depressor

**Figure 2:**
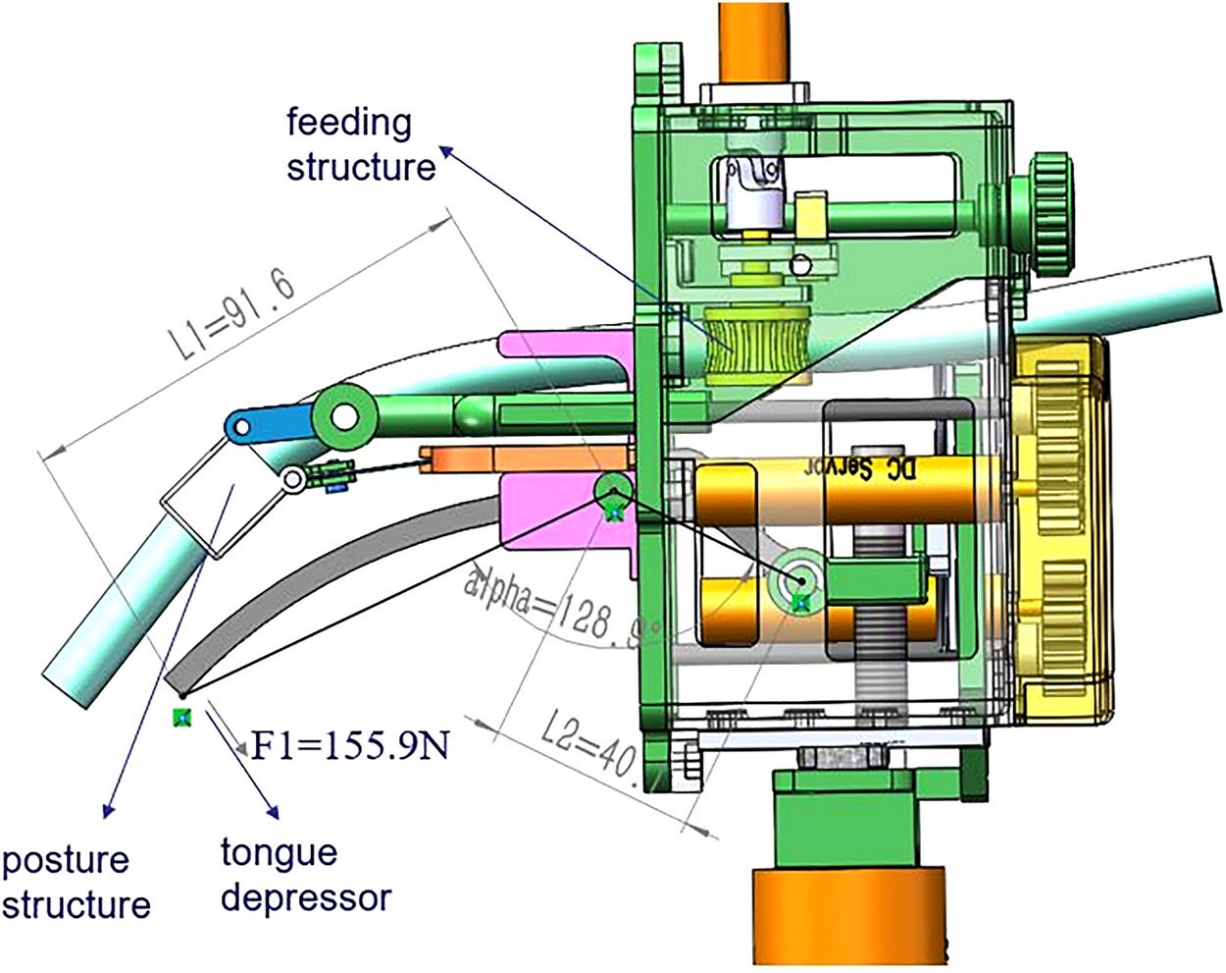
Components of the intubation robot.

An effective tongue depressor can expose the glottis easily and complete the intubation more convenient. As Fig. 2 shows, the primary length of tongue depressor is 91.6 mm (L1), and it can be move forward or backward for 20 mm. The tongue depressor can revolve around the axis for 40 degrees. The max working force of the tongue depressor is 155.9 N (F1) by the present motor, which is sufficient for turning over the epiglottis.

### Posture mechanism

**Figure 3:**
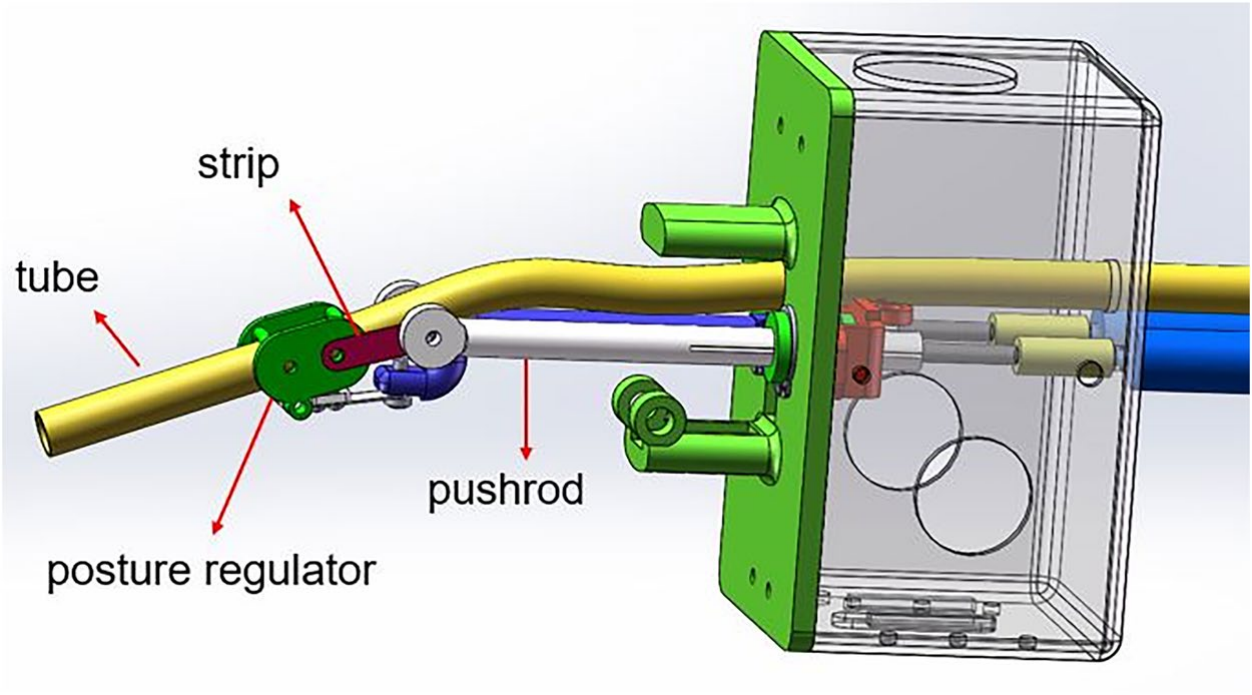
The posture structures.

A 6.0 mm internal diameter endotracheal tube (YiXin Medical, Shanghai, China) is loaded onto the industrial endoscope (H5 Technology, Guangdong, China) to prepare the robot for use. Posture mechanism aims to adjust the posture of the tube’s tip. As Fig. 3 shows, several flexible strips were adopted to fix the posture regulator. The direction of the tube is determined by the different length of the two pushrods.

### Feeding mechanism

**Figure 4:**
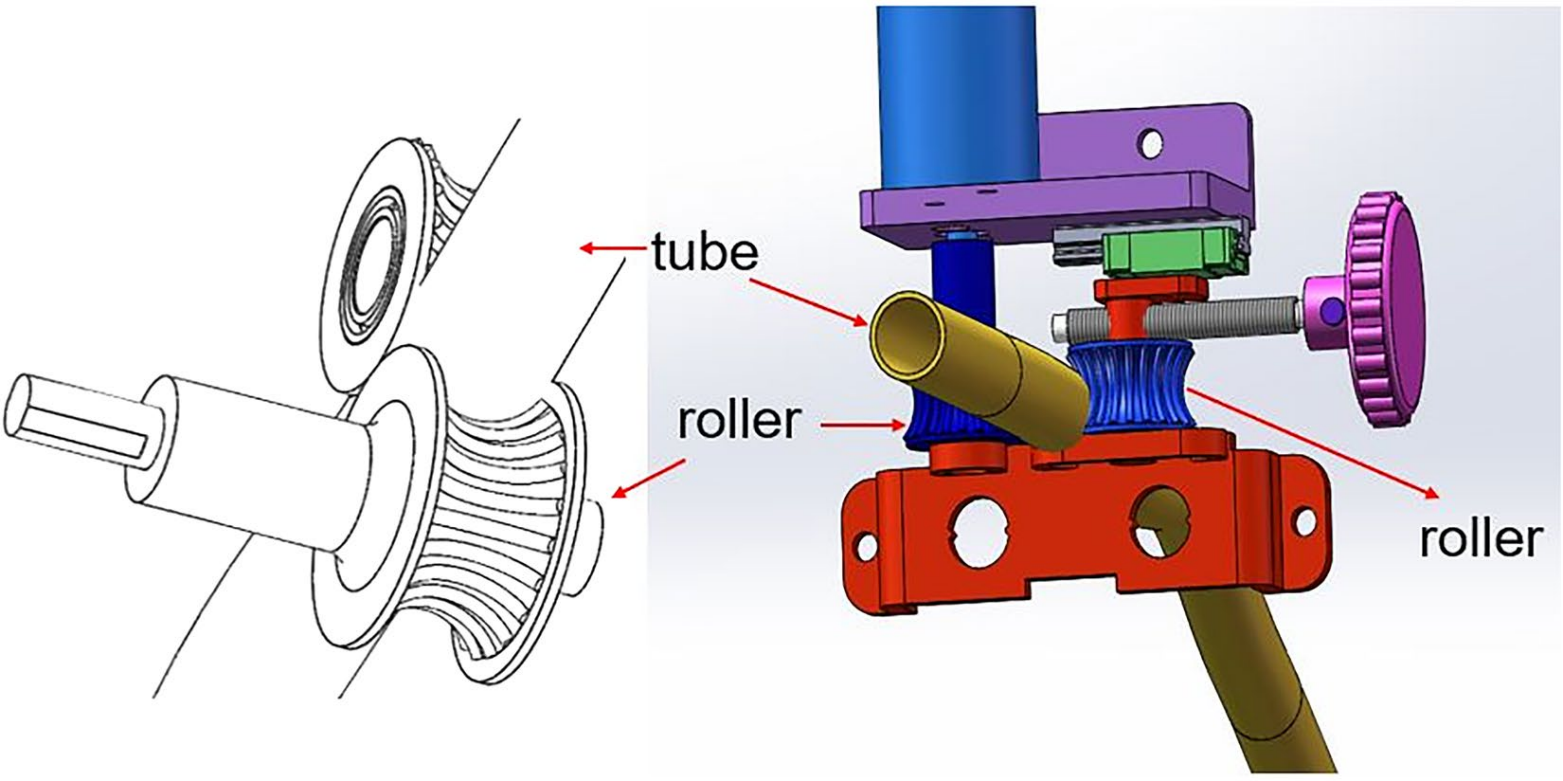
The feeding structures.

Feeding mechanism will push the tracheal tube to move. The feeding structure is composed of two rollers as the Fig. 4 shows. The two rollers rotate clockwise or counterclockwise at the same time, the tube will retract or move forward.

**Figure 5:**
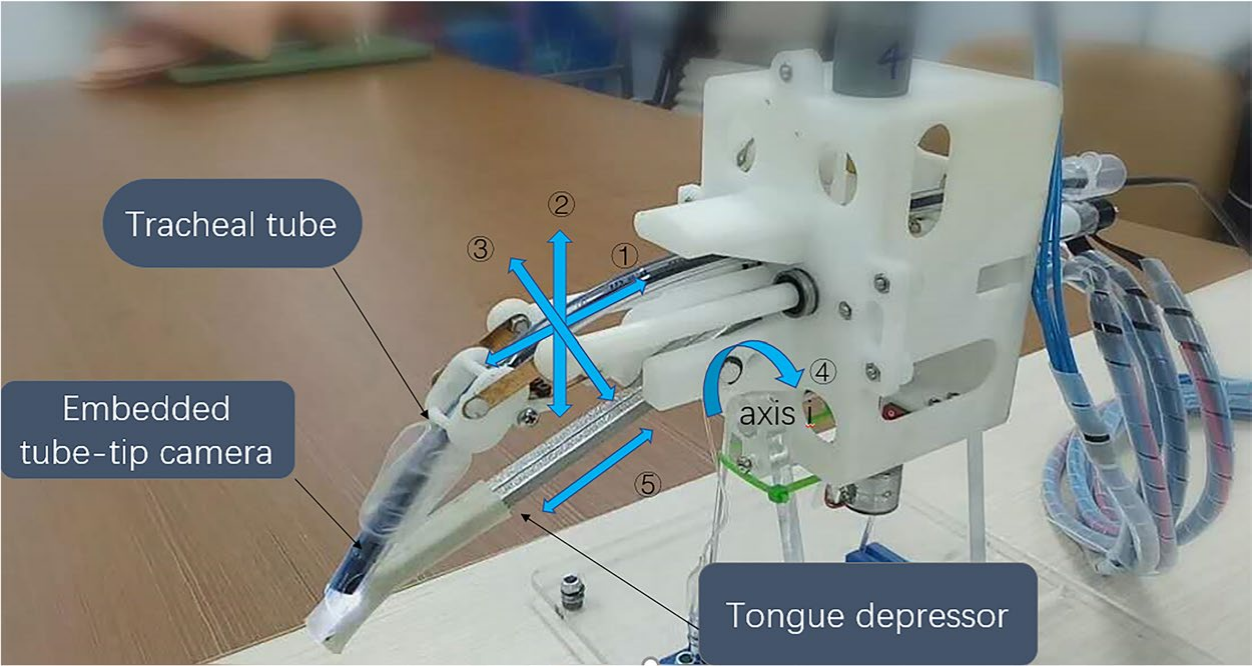
Movements of the intubation robot. There are five types of movements, ① feed and retract of the tube, ② up and down of the tube, ③ left and right of the tube, ④ rotation of the tongue depressor, ⑤ elongate and shorten the tongue depressor.

By these structures, the intubation robot can make five types of movements (Fig. 5): (1) feed and retract the tube, (2) adjust the direction of the tube for up and down, (3) adjust the direction of the tube for left and right, (4) rotate the tongue depressor around the fixed axis i, and (5) elongate and shorten the tongue depressor.

### Supplementary equipment

Special direct laryngoscopes (long, straight blade with a curved tip of 195 mm long) were used for intubation. An electronic sphygmomanometer and a pulse oximeter were used to monitor blood pressure, heart rate and oxygen saturation. A stopwatch was used to record the intubation time.

### Study protocol

Prior to the study, participants received detailed instructions of the robot system and watched video demonstrations of intubation by the system. After these presentations, the participants were requested to practice 5–10 intubations of training on pigs (intubations of training were not performed on those pigs prepared for this study).

Twenty pigs, in accordance with the random number table, were randomly divided into laryngoscope group (n = 10) and robot group (n = 10). There were no differences in body weight between the two groups. In laryngoscope group, the 10 medical students who picked up a pig stochastically and intubated the selected pig by direct laryngoscope. After that, the same 10 medical students turned to the robot group and intubated the pigs by the robot system.

Intubation by direct laryngoscope^4^: An assistant (laboratory worker) held the pig’s mouth open while a medical student held the endotracheal tube in the dominant hand and the laryngoscope in the nondominant hand. The laryngoscope was inserted into the mouth and the tip pressed to the base of the tongue ventrally until the vocal cord opening can be visualized. The student should insert the endotracheal tube slowly until it goes through the glottis. The whole operation was under the supervision of the anesthetist.

**Figure 6:**
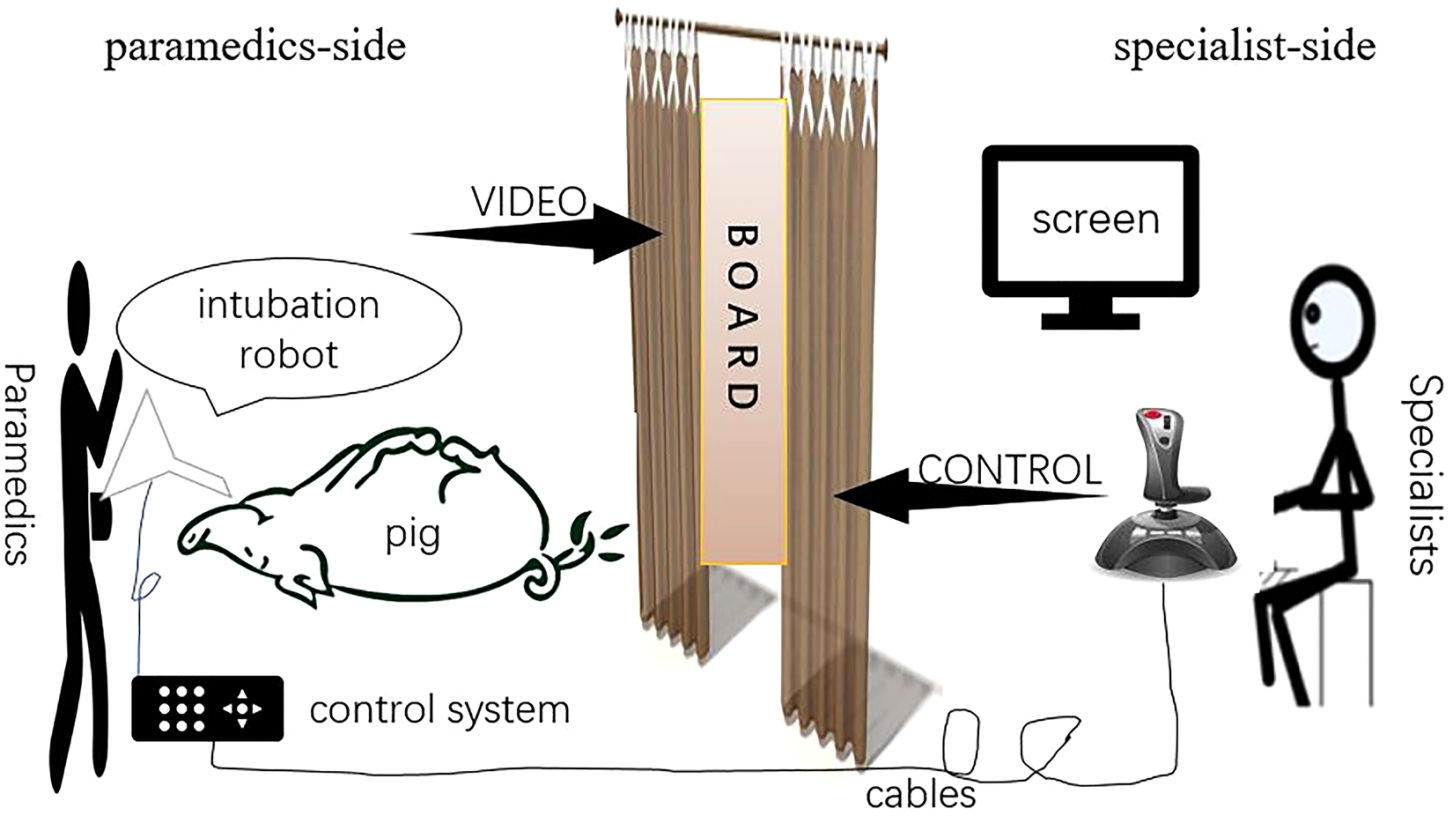
Schematic diagram of the animal experiment. Front emergency spot was simulated with the ‘paramedics-side’. Rear operation team was simulated with the ‘specialist-side’. An opaque board were set in the middle to separate them.

Intubation by the robot system: The intubation robot and the control system were put on ‘paramedics-side’, insulated from the laptop and the joystick on ‘specialist-side’ by a board. The two sides were connected by cables (Fig. 6). An assistant (laboratory worker) held the pig’s mouth open while a medical student inserted the robot into the mouth of pig slowly. Once the lips of mouth reached to the bottom of the robot, the medical student fixed the robot between the upper and lower jaws. Embedded tube-tip endoscopy can deliver images of the mouth structure to the screen from which rear specialist (the anesthetist) who manipulated the joystick can see. At first, the anesthetist should adjust the tongue depressor to the base of the tongue and press down to expose the entrance to the larynx. Then he completed the intubation by controlling the movement and adjusting the direction of the tube immediately after the glottis was exposed by tongue depressor (see supplemental video files).

The successful mark of intubation of both groups was the passage to the glottis of endotracheal tube in the view, ending with inflation of the lungs using a self-inflating ventilation bag. An intubation failure was defined as intubation attempts >3 or intubation time >120 s.

For robot system, the total intubation time, consisted of “frontline time” and “rear time”, was defined from opening the pig’s mouth to removal of the robot. “Frontline time” was defined as the duration of medical students’ operation while “rear time” was defined as the duration of the anesthetist’s operation. For laryngoscope group, “frontline time” was defined as the duration of medical students’ operation. “Rear time” was 0 because the whole work is completely done by medical students.

Intubation time, first-attempt success rate, overall success rate, oxygen saturation, airway complications, the number of optimization manoeuvres required (use of a bougie, external laryngeal pressure, *et al*.), values of systolic blood pressure (SBP), heart rate (HR) 1-min before intubation and 1-min after intubation were recorded.

### Data analysis

SPSS 21.0 software was used to perform data analysis. P values < 0.05 were considered statistically significant. Independent-Samples T-test was performed to compare mean intubation time, elevation of systolic pressure and heart rate for intragroup comparisons. Chi-square test (Fisher’s exact test) was performed to compare first-attempt success rate, overall success rate, number of optimization manoeuvres, and intubation complications.

## Results

There was no death of pigs in the process of intubation. Oxygen saturation was above 95% in all pigs. As is shown in Table 1, the total intubation time by robot system was longer compared with the direct laryngoscope (mean 74.6 ± 2.3 s vs 53.2 ± 3.1 s, P < 0.01). For robot system, the mean value of “frontline time” consumed by medical students was 23.4 ± 0.7 s (Table 2).

**Table 1:** Intubation Data.

Variable	Laryngoscope group (n = 10)	Robot group (n = 10)	P Value
Total time to intubation (s),	53.2 ± 3.1	74.6 ± 2.3	<0.01
First-attempt success rate, n (%)	4 (40%)	8 (80%)	0.17
Overall success rate, n (%)	6 (60%)	9 (90%)	0.30
Intubation complication, n (%)	2(20%)	1(10%)	>0.99
**No. of optimization manoeuvres (%)**			
0	4 (40%)	7 (70%)	0.37
≥1	6 (60%)	3 (30%)	0.37
Elevation of HR (beats/min)	7.7 ± 9.2	17.6 ± 19.9	0.33
Elevation of SBP (mmHg)	16.3 ± 7.8	17.9 ± 5.7	0.89

Values are recorded by mean ± standard deviation.

RRAIS = remote robot-assisted intubation system.

HR = heart rate SBP = systolic blood pressure.

**Table 2:** Intubation time by the robot system.

Pigs	Frontline time (s)	Rear time (s)	Total time (s)
1	24.5	49.3	73.8
2	20.8	43.4	64.2
3	32.4	48.2	80.6
4	27.8	78.1	105.9
5	—	—	—
6	18.2	58.1	76.3
7	26.4	35.6	62.0
8	16.1	40.1	56.2
9	18.8	56.6	75.4
10	25.5	51.6	77.1
Mean(s)	23.4 ± 0.7	51.2 ± 1.6	74.6 ± 2.3

Pig 5 lacking of values was due to failed intubation.

Frontline time was defined as the duration of medical students’ operation.

Rear time was defined as the duration of the anesthetist’s operation.

Four pigs (40%) achieved first-attempt success and six pigs (60%) achieved overall success by laryngoscope while eight pigs (80%) achieved first-attempt success and nine pigs (90%) achieved overall success by robot system. There was no statistically difference in first-attempt and overall success rate between two groups (P = 0.17, 0.30, respectively). However, the absolute numbers of successful intubation by robot system were higher than that by laryngoscope (first-attempt success rate 80% vs 40%, overall success rate 90% vs 60%).

Four failed cases in laryngoscope group were due to excessive attempts or prolonged time. One case failure in robot group was caused by bradycardia (heart rate <30 beats/min) in the course of exposing the glottis, which terminated the intubation process.

Two intubation complications occurred in the process of intubation by laryngoscope, one was mucosal bleeding and the other was esophageal intubation. One intubation complication occurred in robot group was mucosal bleeding.

There was no statistically difference in the number of optimization manoeuvres were required to facilitate tracheal intubation between groups (P = 0.37).

There was no statistically difference in the elevation of HR and SBP (P = 0.33, 0.89, respectively), although figures by laryngoscope were slightly higher than that by the robot system.

## Discussion

We have developed a remote robot-assisted intubation system (RRAIS) and tested it on pigs. We found that participants achieved a higher first-pass rate and overall success rate in absolute numbers by the robot system than with direct laryngoscopy. There was no statistically difference in the elevation of HR, SBP and the occurrence of complications between two groups.

Although total intubation time by the robot system is longer compared with direct laryngoscopy, we found that a shorter time was consumed in the “front” by the robot system compared with direct laryngoscopy (mean 23.4 ± 0.7 s v 53.8 ± 6.2 s s, P < 0.01).

The animal experiment in this study was a simulated wireless intubation. Front emergency spot was simulated with the ‘paramedics-side’ while rear operation team was simulated with the ‘specialist-side’. Pre-hospital paramedics were simulated by medical students and laboratory workers. A long distance was simulated with an opaque board where people on both sides could not see each other absolutely. Remote intubation can be almost fulfilled by creating such a scenario.

Some out-of-hospital diseases, such as cardiac arrest, respiratory failure, traumatic injuries, may result in severe hypoxemia or hypotension^5^. Intubation should be administered immediately when indicated. Therefore, pre-hospital emergency intubation is indispensable in the field for severely ill or injured patients who cannot maintain adequate ventilation.

The skill level of the operator may be a key in determining the efficacy of ETI in acutely ill patients^6^. Ambulance paramedics often lack adequate intubation training and are less familiar with ETI. It is estimated that 47.6% of paramedics had no intubation and 86% had undertaken only two or less intubations over 12-month study period^7^. The failure rates by non-physicians may be 15% higher than the failure rates by physicians^3^. The concept of first pass success is frequently promoted as the goal of emergency intubation in recent years^8^. An increase of airway-related complications is associated with repeated attempts^9^. In addition, intubation on traumatic patients is more complex than non-trauma patients. A meta-analysis shows that the success rate of non-physician clinicians was very low for trauma patients (69.8%) compared with non-trauma patients (87.9%) of pre-hospital intubation^10^.

During pre-hospital rescue, patients will have a potential possibility to lose their lives if on-scene EMTs have no qualifications for ETI or they are inexperienced to perform ETI. Therefore, we developed a remote intubation robot system–RRAIS, so as to provide an alternative intubation arm for pre-hospital ETI. Participants in this study achieved a higher first-pass rate and overall success rate in absolute numbers by the robot system, although there was no statistically difference between two groups, which indicated the robot system may be an effective and feasible approach for EMTs.

**Figure 7:**
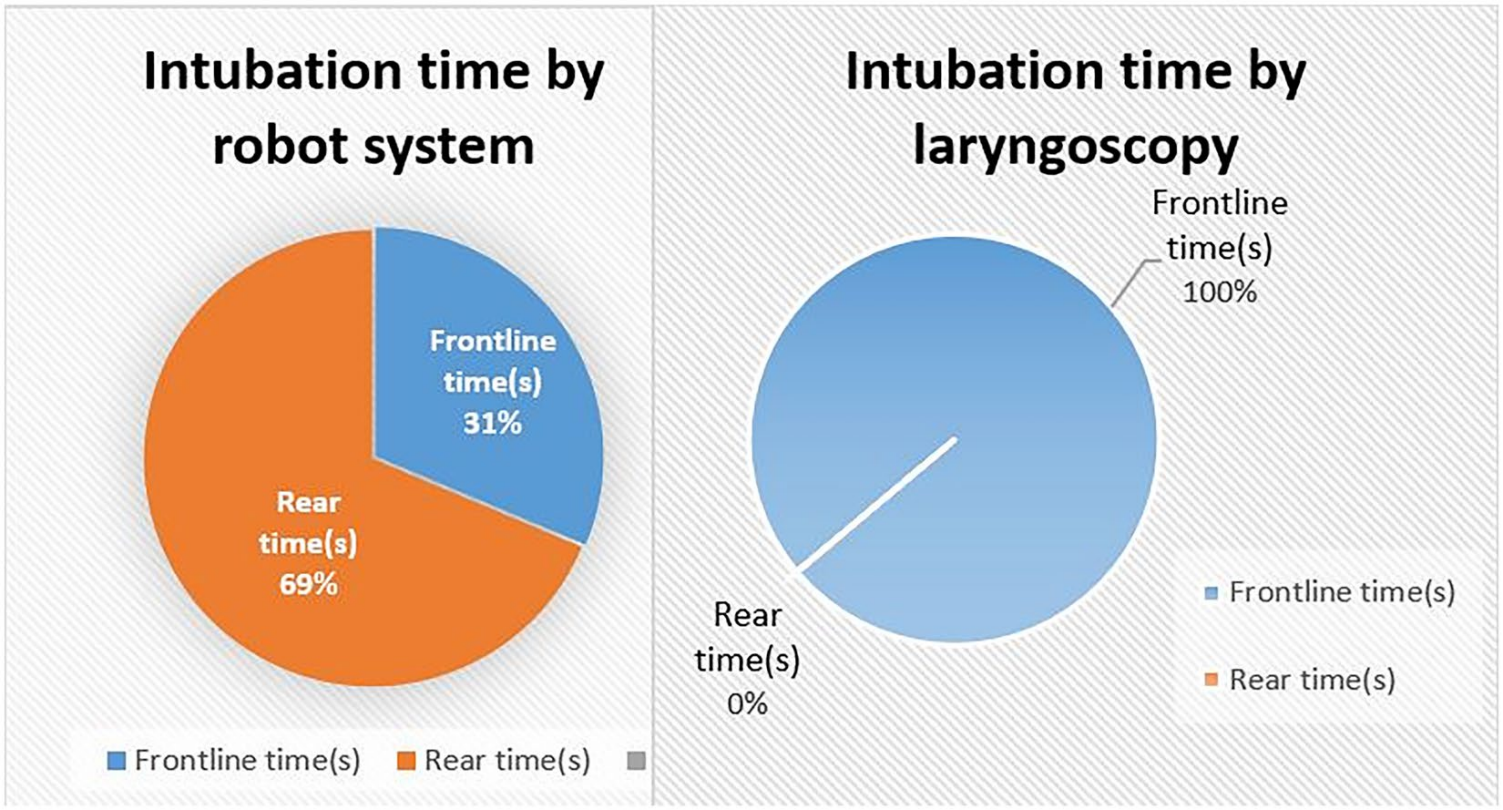
Proportion of frontline time and rear time by two intubation approaches. The proportion of “frontline time” by robot system is 31% while the proportion by laryngoscope is 100%.

RRAIS can divert intubation job from front-line EMTs to rear specialists who have qualifications and richer experience for ETI. Inexperienced EMTs are just required to put the robot into patient’s mouth while the core intubation work is completely done by rear specialists, which reduces the skill demands and frontline time of EMTs. We found that the proportion of “frontline time” by the robot system was significantly lower compared with direct laryngoscopy (31% vs 100%, Fig. 7), which implied that the robot system may be a more convenient and workable approach for EMTs.

RRAIS is aimed at providing out-of-hospital first aid, and should be applied at the following sites and circumstances as we conceive:

1. A moving ambulance. RRAIS can be deployed in each ambulance, which can fulfill the function of first-aid as soon as possible. Ambulances usually play a significant role in natural calamities and traffic accidents. This is the most important application of RRAIS.
2. Battlefields. It can be deployed in the field hospital or tent hospital routinely. It is estimated that 10–15% of battlefield deaths for airway obstruction could be preventable^11^.
3. Nuclear pollution sites, earthquake zones and places with acute infectious diseases. Workers exposed to the above hostile environment are vulnerable to risk factors. For example, a multitude of medical workers got infected in the course of rescue when Severe Acute Respiratory Syndrome (SARS) broke out in China in 2003^12^.
4. Airport, railway station, subway station with a large stream of people. It can be disposed in such locations just like automated external defibrillator (AED) is served for accidents. Not only paramedics but also passers-by can participate in the rescue just required to put the robot into patients’ mouth as we envisage.

Robot-assisted intubations are rare to be studied. Tighe and colleagues completed the robot-assisted intubation in two cases of mannequin with DaVinci Surgical System^13^. Obviously, it is hard to promote this technique in terms of the expense and cumbersome equipment. T.M.Hemmerling successfully developed a robotic system called Kepler intubation system (KIS) for ETI and tested the system in mannequins and patients respectively^14,15^. Hemmerling’s study corroborated the feasibility of robot-assisted tracheal intubation on human.

Some differences exist between KIS and RRAIS. Firstly, KIS is designed for hospital intubation and is expected to assist the anesthesiologist’s hands with less force, higher precision and safety, while RRAIS targets the pre-hospital rescue circumstance, mainly for airway compromise due to certain diseases. Secondly, KIS is so gigantic and clumsy that it is not portable to carry at current stage while RRAIS is easy to popularize due to its small, flexible and portable features. The intubation robot of RRAIS measures approximately 8 inches long * 8 inches wide * 4 inches high.

What matters most is that RRAIS can “transfer” rear specialists to front line, which fulfills the functions of remote medicine. Compared with prior intubation robots, RRAIS is more likely to exert a great influence on emergency and disaster medicine by changing the present rescue model.

We encountered two challenges in mechanical design of the robot system. The first puzzle is whether the robotic arm is necessary for intubation system. An intubation robot using Universal Robot 3 (UR3) was designed by us and was tested it on a manikin in 2015^16^. Compare with Kepler intubation system (KIS), UR3 adopted a smaller and more flexible robotic arm. However, it is also inconvenient to carry this robot system. Now we abandoned the robotic arm for RRAIS and adopted a more compact structure.

The second problem is the necessity of adding the tongue depressor. Tongue depressor is a structure exposing the glottis easily and making the intubation more convenient. We had designed a robot without a tongue depressor in 2016, expecting the tracheal tube to expose the glottis but failed in the experiment^17^. Some articles have demonstrated the feasibility by a camera embedded in the tube tip^18–20^, which gave us an original idea for improving the robot system. Then we adopted an industrious endoscope with tongue depressor and developed RRAIS. RRAIS must be added a tongue depressor due to its compact structure.

We chose pigs as our experimental subjects since there were considerable similarities in the anatomy and physiology between pigs and human^21^. Thus, this device as the figures revealed was designed for pig exclusively. Pig was placed in dorsal recumbency rather than ventral recumbency because it could simulate the process of human intubation, though some articles believe intubation in ventrodorsal position is faster and safer than dorsoventral position^22^. The reason why we firstly conducted the study on animals rather than a manikin was that we could observe the potential changes of vital signs and complications during intubation, which was difficult to observe in manikin experiment. The robot system seemed to be a safe intubation approach because there was not a drastic fluctuation in the changes of HR and SBP in this study.

**Figure 8:**
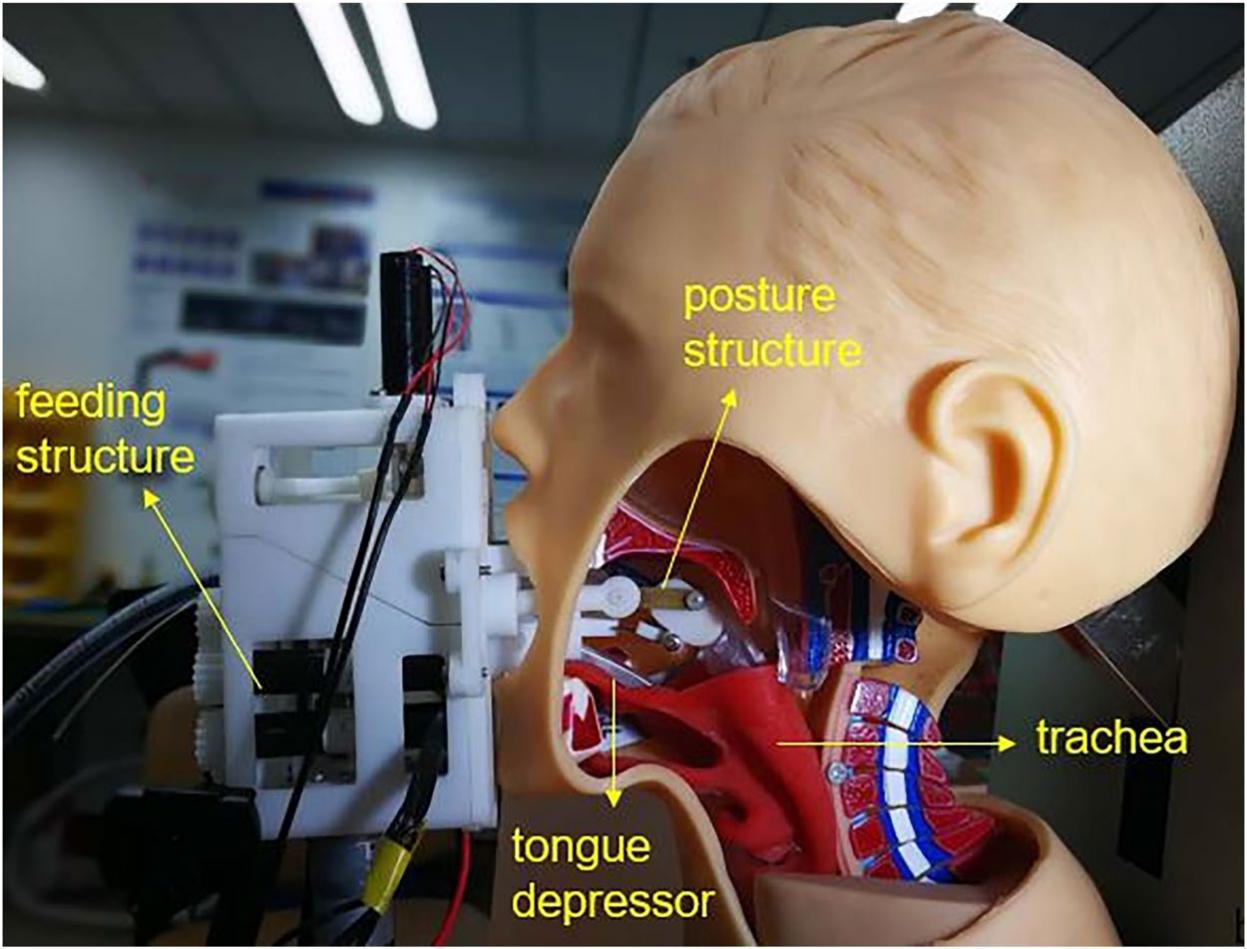
Intubation on manikin. The position of the robot and airway.

The most extraordinary feature of the robot system is wireless long-distance intubation. Next, we plan to focus on the development of the wireless remote-controlled system. Meanwhile, we will create animal models requiring intubation (including cardiac arrest, traumatic injury, and vomit and secretions in the mouth) and apply the system on animal models and manikins (Fig. 8). After completing these, we will try it on patients.

There are several limitations of our research. First and foremost, the evaluation of pig’s intubation complications may be insufficient, which ignored the assessment of some subjective feelings such as postoperative sore throat and hoarseness. In addition, although the tracheas and esophagus of pig have certain structural similarity to those of humans, manikin and human studies are needed to test the robot system in further study.

Secondly, we did not take blood, vomit and secretions into account in the study. It was reported that the most common difficulties influencing pre-hospital ETI were related to blood, vomit, debris and secretions (49.5%)^23^. Simulated hematemesis and vomitus settings should be created on manikin in the next step. An automated tracheal suctioning system has been developed in previous study^24^. A washing and suction device related to RRAIS should be developed in new upgrade product. By this device, it can improve the success rate of pre-hospital intubation by providing clear view when intubation. And also, it can save significant person-hours for nurses and reduce healthcare cost savings after intubation by automated suctioning.

Thirdly, one failed case due to bradycardia was for unknown reasons, may be caused by vagus reflex when the tongue depressor was working. It is essential to provide a force-feedback device in the future.

In conclusion, we have developed a remote robot-assisted intubation system (RRAIS) for pre-hospital ETI and completed a short-distance operation in animal experiment. Future studies will be conducted to improve the functions of RRAIS.

## Electronic supplementary material

animal experiment 1

animal experiment 2

animal experiment 3

animal experiment 4

## Data Availability

The datasets generated during the current study are available from the corresponding author on reasonable request.
